
JOURNAL INFORMATION
==============================
NLM Title Abbreviation: Addict Biol
Journal ID: Addict Biol
NLM Journal ID: 9604935
Journal ID: ADB

Addiction Biology

ISSN: 1355-6215
EISSN: 1369-1600
Publisher: Wiley

ARTICLE INFORMATION
==============================
PMCID: PMC12848586
DOI: 10.1111/adb.70129
Article ID: ADB70129
Article version: 1
Subjects: Issue Information, Issue Information

Issue Information

Electronic publication date: 2026 Jan 28
Publication date: 2026 Feb
Volume: 31
Issue: 2
Electronic Location ID: e70129
Copyright: © 2026 The Authors. Addiction Biology published by John Wiley & Sons Ltd on behalf of Society for the Study of Addiction.
License: This is an open access article under the terms of the http://creativecommons.org/licenses/by/4.0/ License, which permits use, distribution and reproduction in any medium, provided the original work is properly cited.
License URL: https://creativecommons.org/licenses/by/4.0/

Figure count: 0
Table count: 0
Page count: 1

source-schema-version-number: 2.0
cover-date: February 2026
details-of-publishers-convertor: Converter:WILEY_ML3GV2_TO_JATSPMC version:6.6.8 mode:remove_FC converted:28.01.2026

==============================

VOLUME 31 • NUMBER 2 • FEBRUARY 2026

ISSN 1369-1600

### Addiction Biology

Open Access

WILEY

MIX
Paper | Supporting responsible forestry
FSC www.fsc.org
FSC™ C007207

### ADDICTION BIOLOGY

**Editors and Editorial Board**

*Editor-in-Chief*

**Rainer Spanagel, PhD**

*Central Institute of Mental Health CIMH, Institute of Psychopharmacology Heidelberg University D-68159 Mannheim, Germany*

*Editorial Secretary*

**Christine Roggenkamp**

Central Institute of Mental Health (CIMH) Heidelberg University J5D-68159 Mannheim Germany
**E-mail: addiction.biology@zi-mannheim.de**

*Senior Editor for America (Human Studies)*

**Rajita Sinha**

Yale School of Medicine, Department of Psychiatry, 2 Church Street, South New Haven, CT 06519, USA

*Senior Editor for America (Preclinical Studies)*

**Rosana Camarini**

University of São Paulo, Department of Pharmacology, Institute of Biomedical Sciences, Estado de Sao Paulo, Brazil

*Senior Editor for Europe, Africa, Asia and Australia (Human Studies)*

**Antoni Gual**

Psychiatry Department, Neurosciences Institute, Hospital Clínic IDIBAPS 08036 Barcelona, Spain

*Senior Editor for Europe, Africa, Asia and Australia (Basic and Preclinical Studies)*

**Rainer Spanagel**

Central Institute of Mental Health (CIMH), Institute of Psychopharmacology, Heidelberg University D-68159 Mannheim, Germany

**Reviewing Editors**

**Georgy Bakalkin**, *Uppsala, Sweden*
**Christina Barr**, *Maryland, USA*
**Anil Batra**, *Tübingen, Germany*
**Alexis Bailey** - *London, UK*
**David Belin** - *UK*
**Ainhoa Bilbao**, *Mannheim, Germany*
**Stefan Bleich**, *Hannover, Germany*
**Rosana Camerini**, *Sao Paulo, Brazil*
**Bill Carlezon**, *Belmont, USA*
**Roberto Ciccocioppo**, *Camerino, Italy*
**Jeff Dalley** - *UK*
**Veronique Deroche-Gamonet** - *France*
**Dora Duka**, *Brighton, UK*
**Fernando Rodríguez de Fonseca**, *Malaga, Spain*
**Johan Franck**, *Stockholm, Sweden*
**Teresa Franklin**, *Philadelphia, USA*
**Jean-Antoine Girault**, *Paris, France*
**David Goldman**, *Rockville, USA*
**Katia Gysling**, *Santiago, Chile*
**Christian Heidbreder**, *Richmond, USA*
**Stephen C. Heinrichs**, *Chestnut Hill, USA*
**Andrew Holmes**, *Bethesda, USA*
**Daniel Hommer**, *Bethesda, USA*
**Yasmin Hurd**, *New York, USA*
**Petri Hyytiä**, *Helsinki, Finland*
**Yedy Israel**, *Santiago, Chile*
**Falk Kiefer**, *Mannheim, Germany*
**Brigitte Kieffer**, *Illkirch, France*
**Hank Kranzler**, *Philadelphia, USA*
**Mary Jeanne Kreek**, *New York, USA*
**Andrew Lawrence**, *Melbourne, Australia*
**Anne Lingford-Hughes**, *Bristol, UK*
**Hilary Little**, *London, UK*
**David Lovinger**, *Rockville, USA*
**Lin Lu**, *China*
**Rafael Maldonado**, *Barcelona, Spain*
**Christian Müller**, *Erlangen, Germany*
**M. Foster Olive**, *Charleston, USA*
**Pier Vincenzo Piazza**, *Bordeaux, France*
**Ryszard Przewlocki**, *Cracow, Poland*
**Dorit Ron**, *San Francisco, USA*
**Scott Russo**, *New York, USA*
**Andrey Ryabinin**, *Portland, USA*
**Susan Schenk** - *New Zealand*
**Carla Sanchis-Segura**, *Castello, Spain*
**Gunter Schumann**, *London, UK*
**Yavin Shaham**, *Baltimore, USA*
**Mohammed Shoaib**, *Newcastle, UK*
**Rajita Sinha**, *New Haven, USA*
**Michael Smolka**, *Dresden, Germany*
**Wolfgang Sommer**, *Mannheim, Germany*
**Dai Stephens**, *Brighton, UK*
**Tsutomu Suzuki**, *Tokyo, Japan*
**Karen Szumlinski**, *Santa Barbara, USA*
**Boris Tabakoff**, *Aurora, USA*
**Todd Thiele**, *Chapel Hill, USA*
**Thomas Tzschentke**, *Aachen, Germany*
**Louk Vanderschuren**, *Utrecht, the Netherlands*
**Elise M. Weerts** - *USA*
**Friedbert Weiss**, *La Jolla, USA*
**Reinout Wiers**, *Amsterdam, the Netherlands*
**Gerald Zernig**, *Innsbruck, Austria*
**Andreas Zimmer**, *Bonn, Germany*
**Ulrich Zimmermann**, *Dresden, Germany*

**Open Access and Copyright**

All articles published by *Addiction Biology* [from Jan 1, 2024] are fully open access: immediately freely available to read, download, and share. All articles [from Jan 1, 2024] are published under the terms of a Creative Commons license.

Copyright on any research article published by *Addiction Biology* is retained by the author(s).

Further information about open access licenses and copyright can be found at: https://authorservices.wiley.com/author-resources/Journal-Authors/licensing/licensing-info-faqs.html#4. Information on the licenses used by *Addiction Biology* can be found at: https://onlinelibrary.wiley.com/journal/13691600.

**Disclaimer**

The Publisher, the Society for the Study of Addiction and Editors cannot be held responsible for any errors in or any consequences arising from the use of information contained in this journal. The views and opinions expressed do not necessarily reflect those of the Publisher, the Society for the Study of Addiction or Editors, neither does the publication of advertisements constitute any endorsement by the Publisher, the Society for the Study of Addiction, Editors, or Authors of the products advertised.

Wiley Open Access articles posted to repositories or websites are without warranty from Wiley of any kind, either express or implied, including, but not limited to, warranties of merchantability, fitness for a particular purpose, or non-infringement. To the fullest extent permitted by law Wiley disclaims all liability for any loss or damage arising out of, or in connection with, the use of or inability to use the content.

For submission instructions and all other information visit: https://onlinelibrary.wiley.com/journal/13691600
